# Individualized approach to primary prevention of substance use disorder: age-related risks

**DOI:** 10.1186/s13011-020-00300-7

**Published:** 2020-08-14

**Authors:** Eric Afuseh, Caitlin A. Pike, Ukamaka M. Oruche

**Affiliations:** 1 Indiana University School of Nursing, Indianapolis, IN USA; 2 Indiana University Purdue University Indianapolis, Indianapolis, IN USA

**Keywords:** Substance use disorder, Primary prevention, Opioid, Substance misuse, Risk factors

## Abstract

**Background:**

The misuse of legal and illegal substances has led to an increase in substance use disorder (SUD) in the United States. Although primary prevention strategies have been successfully used to target chronic physical diseases, these strategies have been less effective with SUD, given misconceptions of SUD, shortages in behavioral health professionals, and the population-based focus on specific substances. A developmental approach to the identification and primary prevention of SUD that does not fully rely upon behavioral health workers is needed. The purpose of this paper was to examine age related risk factors for developing SUD and present a novel individualized approach to SUD prevention.

**Methods:**

A literature search was conducted to identify risk factors for SUD among children, young adults, adults, and older adults. We searched CINAHL, PsycINFO, and PubMed between the years 1989–2019, and extracted data, analyzing similarities and differences in risk factors across life stages. Broader categories emerged that were used to group the risk factors.

**Results:**

More than 370 articles were found. Across all age groups, risk factors included adverse childhood experiences, trauma, chronic health diseases, environmental factors, family history, social determinants, and grief and loss. Despite the similarities, the contextual factors and life challenges associated with these risks varied according to the various life stages. We proposed an approach to primary prevention of SUD based on risk factors for developing the disease according to different age groups. This approach emphasizes screening, education, and empowerment (SEE), wherein individuals are screened for risk factors according to their age group, and screening results are used to customize interventions in the form of education and empowerment. Given that trained persons, including non-healthcare providers, close to the at-risk individual could conduct the screening and then educate and mentor the individual according to the risk level, the number of people who develop SUD could decrease.

**Conclusions:**

The risk factors for developing SUD vary across the various life stages, which suggests that individualized approaches that do not overtax behavioral healthcare workers are needed. Using SEE may foster early identification and individualized prevention of SUD.

## Introduction

Substance use disorder (SUD) is a deadly disease that affects people across all age groups [1]. SUD often begins as a pattern of substance misuse characterized by using psychotropic substances without a prescription or in a manner other than prescribed (e.g., using substances more frequently or for a longer duration than indicated by prescribing guidelines) [2]. Each day in the US, over 4300 adolescents, aged 12 to 17 years, use substances for the first time [3]. The number of people who eventually develop SUD after using a substance for their first time is unknown. The most frequently misused substances include nicotine, alcohol, opioids, central nervous system depressants (e.g., benzodiazepines), stimulants (e.g., Ritalin), marijuana, anabolic steroids, cocaine, methamphetamine, amphetamines (e.g., Adderall), MDMA (e.g., ecstasy), hallucinogens, and inhalants [4, 5]. Misuse of both legal and illegal substances has led to an increase in SUD in the United States (US) [6]. Although there are effective, evidence-based treatments for SUD, most individuals suffering from SUD do not receive treatment until advanced stages of the disease [7].

It was once widely believed that SUD was the result of a choice or character flaw. However, SUD is a mental illness that should be treated like any other chronic physical health conditions [8, 9]. Primary prevention, which involves strategies to avoid the development of the disease, has been an effective strategy for chronic physical health problems (e.g., diabetes and cardiovascular diseases) [10, 11]. Although risk and protective factors for developing SUD are well-documented, the public health system has not effectively leveraged primary prevention strategies to identify or flag individuals at-risk for SUD [12–14]. The Substance Abuse and Mental Health Administration (SAMHSA) provides guidelines on the assessment of the community, but not risk factors for individuals [15]. Currently, the mental health infrastructure in the United States is not sufficient to meet the needs of its population, in part due to shortages in behavioral health professionals [16].

Federal, state and local agencies have put resources and processes in place to address SUD at different levels of the disease continuum [17–19]. Many of these agencies use primary prevention strategies, such as public health announcements or community education programs, to address specific substances (e.g., opioids, nicotine, or alcohol) at the population level [19, 20]. However, these strategies do not fully account for: 1) the unique developmental factors and life stressors in different age groups, 2) the potential misuse of more than one substance at a time, and 3) multiple risk factors for developing SUD.

Therefore, there is an urgent need to identify innovative ways to meet the needs of a growing number of patients with SUD. Identifying age-related risk factors for the development of SUD is important in recognizing individuals at risk. A comprehensive list of risk factors within each age group is needed to develop age-specific screening tools that can identify individuals at-risk early. Given the shortage of behavioral health professionals, screenings can be performed by trained non-medical personnel with knowledge about individuals, their families, and friends. Using a developmental approach to risk assessment will also allow clinicians to develop tailored interventions, education, and support systems for individuals at-risk for developing SUD.

Given that primary prevention strategies may decrease the number of individuals who develop SUD, we proposed a risk mitigation approach to the primary prevention of SUD based on age groups, which will allow clinicians to consider developmental stressors unique to each group. First, we searched the literature to identify risk factors for developing SUD and grouped them in broader categories under four life stages (children, young adults, adults, and older adults). Then, we described an approach for primary prevention of SUD, which emphasizes 1) screening based on the identified risk factors in different age groups, and 2) developing individualized age-appropriate interventions, using education and empowerment, to differentially address age-related motivations for misusing substances.

## Methods

We conducted a search of the literature to identify risk factors for developing SUD. The search was done in CINAHL, PsycINFO, and PubMed for articles published from 1989 to 2019, which yielded over 370 articles. In reviewing the articles, broader categories of the risk factors emerged (e.g., chronic diseases, Trauma and environmental factors). The broader categories of risk factors were then used to identify their impact across the different life stages (children [< 18 years], young adult [18–25 years], adult [26–64 years], and older adults [> 65 years]. We identified similarities and differences across age groups and then selected reoccurring themes for further in-depth review. Articles published from 2005 to 2019 regarding the broader identified categories were included from the search. Several articles with similar content about SUD risk factors and/or published after 2005 were excluded, and a hand-search was conducted to fill in any remaining gaps.

## Results

Table 1 shows identified risk factors for developing SUD by life stages or age groups. We first present here a summary of the risk factors within each age group. Next, we discuss the recurring risk factors across the age groups using examples from the literature.

**Table 1:** Risk Factors for Developing Substance Use Disorder by Age Groups

Children *Less than 18 years*	Young Adults *18 years – 25 years*	Adults *26 years – 64 years*	Older Adults *Over 65 years*
ACEs	ACEs	ACEs	ACEs
Physical or Emotional Trauma	Physical or Emotional Trauma	Physical or Emotional Trauma	Physical or Emotional Trauma
Chronic Health Problems	Chronic Health Problems	Chronic Health Problems	Chronic Health Problems
Environmental Factors	Environmental Factors	Environmental Factors	Environmental Factors
Family History	Family History	Family History	Family History
Social Determinants	Social Determinants	Social Determinants	Social Determinants
Grief and Loss	Grief and Loss	Grief and Loss	Grief and Loss
	Higher Education	Higher Education	
	Military Service	Military Service	
		Healthcare Professionals	

### Summary of risk factors by age group

#### Children (< 18 years)

Among children and adolescents, there were several risk factors for developing SUD, including adverse childhood experiences (ACEs) and trauma [21–23]. Environmental risk factors included peer pressure, participation in organized athletics, and a family history of substance misuse [24–26]. Demographic risk factors for SUD included race, sexuality, gender identity, and socioeconomic status [27–30].

#### Young adults (18–25 years)

For young adults who already have childhood risk factors (e.g., ACEs), the added stress of adulthood can place them at risk for misusing substances. Latent family history, lack of positive parental role models, employment, and academic stress also can increase the likelihood of young adults misusing substances [26, 31–33]. Additionally, receiving potentially addictive prescription medications for the first time (e.g., for minor surgical procedures) put young adults at risk for SUD [34, 35].

#### Adults (26–64 years)

The major stressors for this age group are related to family life and career. Different careers are associated with SUD in adults, including high-stress jobs and heavily physical jobs (e.g., healthcare, military service, and law) [36–38]. Professional athletes in this age group, who perform both high stress and extremely physical jobs, also are at an increased risk of SUD [39].

#### Older adults (> 65 years)

While many of the risk factors (e.g., posttraumatic stress disorder and ACEs) in younger age groups are present in older adults, they also experience unique age-related risk factors for SUD such as experiencing grief and loss more frequently, due to deaths among family and friends [40]. This sense of loss can also lead to physical and social isolation which then leads to SUD [41]. Their greater tendency for chronic physical illnesses (e.g., arthritis and other chronic pain conditions), increase the likelihood of misusing substances to relieve pain [42].

### Recurring risk factors across age group

#### Adverse childhood experiences (ACEs)

Research suggests that individuals who have ACEs are at risk of developing SUD across the lifespan [21]. According to the Children’s Bureau, any form of abuse, neglect, or other traumatic experience that occurs to persons younger than 18 years of age constitutes ACEs [43]. ACEs have been linked to risky health behaviors, including substance use [21, 44]. Additionally, ACEs have been associated with chronic disease, which was an independent risk factor for SUD [45–47]. ACEs have been found to contribute to behavioral health conditions which often co-occurred with SUD (e.g., depressive disorders and anxiety disorders), and unsurprisingly, ACEs have also been linked to increased suicidality [22, 48]. A recent study also found that children who experienced ACEs and were in foster care were at increased risk for SUD later in life [23].

#### Physical or emotional trauma

Trauma has been associated with SUD across all age groups [35, 39, 41, 49]. During adolescence, trauma may be physical (e.g., injuries due to an accident) or emotional (e.g., harassment or hazing). Studies have shown that bullying (physical, verbal, or cyber) can contribute to physical and emotional trauma which then leads to SUD [27, 50]. Teenagers who were bullied were more likely to experience depression, which made bullying a secondary risk factor for SUD [27, 51]. Earnshaw et al. found that elementary students who experienced bullying in fifth grade were more likely than their non-bullied peers to use substances by tenth grade [51]. The tenth graders who used substances also were more likely to be identified as depressed in seventh grade, which then leads to substance misuse later in life [51]. Depression and poor coping skills due to emotional trauma were strong indicators of potential substance misuse among young adults [33]. Minor surgical procedures (e.g. wisdom tooth extraction) can put individuals at risk for developing SUD, regardless of age [34, 52]. For example, among opioid-naïve patients, if an opioid prescription was filled because of wisdom tooth extraction, patients were more likely to continue using opioids after recovery from the procedure [34]. Professional athletes also have a high risk of SUD due to pain from sports-related trauma and the risk persists even after retirement [39].

#### Chronic health problems

Chronic mental illnesses (e.g., depression, PTSD, attention deficit disorder, oppositional defiant disorder) and/or physical health problems (e.g., diabetes, rheumatic conditions, and inflammatory bowel disease) are risk factors for substance misuse leading to SUD in children [53, 54]. On average, mood and anxiety disorders appeared 3 years before the first diagnosis of SUD, particularly when they co-occurred with ACEs [22]. This means the risk factors for SUD can have a cumulative effect on developing SUD and early detection could help with prevention. In youth diagnosed with chronic mental illness (e.g., PTSD), being of certain demographics (e.g., female and/or Latino), increased the risk of SUD especially opioids [53].

For chronic physical conditions, studies suggest that chronic pain at any life stage increases the likelihood of opioid misuse and thus SUD [42]. Using the National Survey on Drug Use and Health (NSDUH), Han and colleagues found that 91.8 million (37.8%) noninstitutionalized US adults used prescription opioids in 2015, and of these, 12.5% reported misuse was motivated by the desire to relieve physical pain [2]. Cancer survivors were at risk of developing opioid misuse because of easy access to pain medications associated with their treatment particularly among older adults [55]. In post-surgical pain management, patients are more likely to develop SUD the longer an opioid is prescribed, regardless of the dosage [56].

#### Environmental factors

Peer pressure was identified as a major risk factor for opioid misuse among high school students in recovery settings [24]. Several environmental factors (e.g., access to illegal drugs, participation in organized athletics, and off-campus housing), influence opioid misuse among college students, which can lead to SUD [26, 32, 33]. Participation in high-contact athletics (e.g., football, hockey, and soccer), increased the risk of physical injuries (e.g., fracture, muscle sprain/strain, or concussion), which may result to substance misuse for pain relieve [25, 57]. Athletes who experienced injuries were found to be at increased risk of non-prescription opioid misuse [35].

#### Family history

Family history as a risk factor for developing SUD can be a combination of genetic inheritance and/or home environmental factors (e.g., substance exposure/availability at home, and normalization of substance use by family members) [58–60]. A family history of substance misuse or SUD substantially increases the likelihood that a child will use substances [60]. Availability of illicit substances in the home during childhood and adolescence, increased the likelihood of developing SUD as a young adulth [26]. Mother-child relationships was shown to influence opioid misuse among young adults, with low mutual attachment associated with a higher risk of opioid misuse [61]. Parents may be unaware that their children misuse family members’ prescribed substances and that this misuse by the children lead to SUD [62].

#### Social determinants

The risk factors under social determinants can be either demographic (e.g., race, sexuality, and gender identity) or socioeconomic (e.g., income and level of education) [28–30, 63]. For example, compared to white adolescents of the same age, Latino and black adolescents were at increased risk for opioid misuse, with Hispanic adolescents twice as likely as white peers to misuse opioids [63]. In addition, Native American children who had a high perceived frequency of racial discrimination were more likely to misuse prescription drugs [64]. Considering gender identity as a risk factor, transgender youth were more likely to develop SUD than their cisgender peers [29]. Among young adults, homelessness and certain gender identity (e.g., lesbian, gay, bisexual, transgender, or queer) also place the individual at an increased risk of developing SUD [28, 30, 31]. Other socioeconomic factors (e.g., unemployment and low level of education) can lead to substance misuse [2, 65, 66].

#### Grief and loss

Across all life stages, experiencing grief and loss (e.g., divorce, death of a close family or friend) can lead to substance misuse [40, 67, 68]. For older adults, loss of a close relative or friend leads to depression and ultimately SUD especially in the presence of other factors (e.g., social isolation, physical immobility, and loneliness) [69, 70]. Elderly people who do not maintain their own needs (e.g., personal hygiene, nutrition, and basic sanitation) due to self-neglect were at increased risk of prescription drug misuse, including opioids [41].

#### Higher education

Several factors (e.g., disposable income, availability, academic pressure, and poor judgment) place college students of any age at a greater risk for SUD [32, 71, 72]. In particular, college women who experienced trauma and diagnosed with PTSD reported higher rates of opioids misuse leading to SUD [73]. Students in certain healthcare programs (e.g., nurses, and anesthesiology residents), were at increased risk for developing SUD with opioid misuse more common [74, 75]. Students with poor program performance and off-campus students were more likely to misuse opioids than their higher performing, on-campus peers [76].

#### Military service

Military service members and veterans are at an increased risk of SUD due to several factors (e.g., PTSD, chronic pain, and mental health) [37, 77]. Military personnel who killed in combat were more likely to have mental health problem and develop SUD especially alcohol misuse [37, 77]. Similar with non-veterans, social determinants (e.g., younger age, lower income, and fewer years of education) were associated with opioid misuse among veterans [78].

#### Healthcare professionals

Healthcare professionals (e.g., pharmacists, nurses, dentists, and anesthesiologists) have a higher risk of opioid misuse primarily due to the access and availability of substances in their professions, as well as the risk of work-related injury [36, 79]. Long work shifts and physical injury on the job were specifically identified as SUD risk factors for nurses [80]. Among all healthcare workers, the lack of SUD awareness education in school translated into increased risk in the profession, as did the tendency to view SUD as a personal and moral failure within the profession, despite all evidence to the contrary [36, 80, 81]. Jobs related to anesthesia were particularly at risk of opioid misuse compared to other types of healthcare workers [82–84].

## Discussion

Although current prevention strategies for SUD address the problem at a community level, these strategies have overlooked the importance of individual risk factors for developing SUD at different life stages. Upon examining the literature, we found that for each age group (children, young adult, adult, and older adult) certain unique developmental and life stressors predispose them for developing SUD. The risk factors that were common across all age groups included ACEs, Trauma, chronic health problem, environmental factors, family history, social determinants, grief and loss. Given these findings, we recommend an approach to prevention of SUD that takes into account risk factors in each age group. The individuals who can potentially develop SUD are those who have never used substances, previous users of substances, and current users of prescriptions with a legitimate need.

Using this approach, we recommend the following 3 steps. First, develop a list of evidence-based risk factors for developing SUD in each age group. Second, use the list of risk factors to develop and validate screening tools that are specific to each developmental group. These tools could measure the cumulative effects of risk factors for individuals in a particular age group. The risk factors can be quantified such that the total score for individuals can be used to identify those at low and high risk for developing SUD. Third, develop intervention strategies based upon the screening results. The interventions can take different forms, such as education, empowerment through mentoring, social media targeted communications, or workplace orientation information. Quantifying risk factors also will help clinicians and researchers design primary prevention interventions tailored to individuals’ risk levels.

This innovative approach can become the standard for individualized primary prevention of SUD, namely screening, educating, and empowering (i.e., SEE). Using validated tools to screen helps identify individuals at-risk while educating and empowering can be used for intervention or prevention. Below, we offer an example of how this primary prevention approach for SUD using an age screening tool can be applied in practice for a specific age group.

A 15-year-old Latino-American female who was raised in foster care, sexually abused at 13 years of age, and identifies as a lesbian, would have a higher score when screened, indicating a greater risk of developing SUD than her peers of the same age without similar risk factors. An education strategy for this 15-year-old could include providing materials about SUD consistent with her level of maturity and understanding. Empowerment could consist of pairing this student with a positive role model like a favorite teacher. The role model would periodically talk with this at-risk person about the benefits of positive behavior and avoidance of risky behavior. The role model could be proactive and identify potential problems, reinforce positive behavior, or initiate appropriate interventions.

There are three advantages to using the SEE approach. First, this approach is not limited to healthcare workers in the community. Anyone, including parents, educators, employers, and even close friends, can initiate the screening process. Second, this approach is less likely to require structured counseling by unfamiliar people, given that people the at-risk individual knows and trust can implement educational and empowerment strategies. Last, formal, professional counseling can be used to augment the process in more complicated, high-risk situations.

### Limitations

The scope of the literature review may have been limited by our key words used in the search and the databases use. Therefore, additional searches using all possible key words and databases may reveal more risk factors for inclusion in the development of a screening tool.

## Conclusion

SUD is a public health crisis in the US, which affects people across all age groups. Similar to other chronic diseases, primary prevention is a vital component in the fight against SUD. Identifying age-related risk factors for the development of SUD is important in recognizing individuals at risk. Putting the population into various age groups allows for the identification of unique developmental factors and life stressors. A comprehensive list of risk factors within each age group is needed to develop age-specific evidenced based screening tools and customize appropriate individual interventions. Screening can be performed by trained non-medical personnel with knowledge about individuals and their families. Using the model of SEE allows for the early identification of individuals at risk. It also helps clinicians and researchers create age-appropriate supports for individuals at-risk for developing SUD. With this model, at-risk individuals are provided with information to create awareness and encourage avoidance of substance misuse.

## Data Availability

Not Applicable.

## Abbreviations

ACEs: Adverse Childhood Experiences
NFL: National Football League
NSDUH: National Survey on Drug Use and Health
PTSD: Post- Traumatic Stress Disorder
SAMHSA: Substance Abuse and Mental Health Services Administration
SEE: Screening, Educating and Empowering
SUD: Substance Use Disorder
US: United States
